# Lycoperdon for Alveolar Hæmorrhage

**Published:** 1895-06

**Authors:** C. Brewster

**Affiliations:** Montreal.


ARTICLE II.

### LYCOPERDON FOR ALVEOLAR HÆMORRHAGE.

BY C. BREWSTER, L. D. S., MONTREAL.

Read at Vermont State Dental Association.

I have been requested this evening to address to you a few words on the subject of lycoperdon as a styptic for arresting alveolar hæmorrhage. Like a great many other things, this material is *not* "new under the sun." It is a growth of nature, to whom we owe so many of our best remedies, and is found in most countries. It is one species of a very large family, however; and though most of the different kinds of the lycoperdon are useful as styptics, there is one special kind that is better than all others. It, however, takes considerable experience to discern the good from the indifferent. Lying in the fields for any great length of time, exposed to the various changes of atmosphere, it is subject to a certain deterioration which is only detected by careful examination. Fully a fourth of what I have had collected for me I have been obliged to throw away as useless.

As quinine in its own department—itself one of Nature's own special remedies—stands pre-eminent for its curative powers over all competitors; so, too, lycoperdon will be found supreme in its curative powers over all other styptics for the arrest of alveolar hæmorrhage. One of its peculiarities is its *healing power*. Plugging the alveolar cavity with any of the other known styptics, such as perchloride of iron, tannin and many other well-known remedies, too numerous to mention, is invariably followed, after the bleeding has been arrested, by much inflammation and severe pain. When, however, we use the lycoperdon for plugging the cavity, we find the very opposite effect is produced, the wound instead of presenting an angry, irritated appearance, and healing up very slowly, shows every inclination to heal by first intention, and not only that, but it shows a decided tendency to heal more rapidly than under ordinary circumstances. This can be easily proved by taking a case where two teeth have been extracted from the same mouth at the same time, and plugging one of them with lycoperdon and leaving the other to the usual process of nature. It will be found that the cavity that has been plugged will heal the fastest. Fortunately alveolar hemorrhage is not a very frequent occurrence, but *unfortunately* it has a bad habit of springing itself on a dentist at the most unexpected times, and often when he is not prepared with the proper remedies to meet the case, his perchloride of iron has evaporated or looks as though it has gone bad, his tannin he can't find, and so on, and so on. Somehow these bleeding cases have a bad habit of coming on in the night, or some other awkard time.

I have forgotten where I met the historical statement that when Cæsar landed in Britain the soldiers staunched their wounds with the puff-balls they found in the fields. In 1853, Dr. Benjamin Ward Richardson experimented with the form of fungus called lycoperdon giganteum, having been led to do so by witnessing its use to stupefy bees before robbing hives, a custom in use centuries ago in England. Dr. Richardson found that, exposed to heat the fumes produced anæsthesia, and from 1853 to 1860 he thus narcotized more than one thousand animals, also making himself unconscious by its use. He recommended its revival as a styptic in alveolar hæmorrhage, but, for some reason unknown to me, it was not popularized. I am disposed to believe that it may have been due to the fact that the species of fungus with which he experimented, the lycoperdon giganteum, was not the best; and it is possible, too, that the decrease of want of contractility in the blood-vessels, and especially of alveolar hæmorrhage, due to the more general use of fruits and better hygienic knowledge, made the matter of less importance to him than it was thirty years ago. There are still, however, very frequent cases of hæmorrhage. In all cases of anæmia, where the fibrin is in inefficient solution, and the blood itself is of feeble coagulating power, the vascular trunk will have feeble, contractile power, and hæmorrhage is apt to occur after tooth extraction.

Of course in marked hæmorrhagic diathesis, or when excessive bleeding depends upon some previously existing disease, it is wise to avoid or defer surgical operations in the mouth, if possible. The regulation of diet and the constitutional precautions are outside the province of the dentist, and should be relegated to the family physician.

However, when we meet with persistent hæmorrhage in our office, one of the first things to do is to discover if the flowing blood is a blood that will coagulate. If a little is caught in a spoon, and the fibrin is seen to clot in three minutes, the vascular or mechanical cause will disappear of itself. But if the blood will not clot, we can rarely, if ever fail by plugging the socket with the lycoperdon. I have seen failures from perchloride of iron, tannin, and the other styptics of the pharmacopœa. I have known perchloride of iron to be used at the boiling-point with no effect; and not long ago a case occurred in Montreal, one of spontaneous bleeding from the gums, in which no extraction had been performed, and notwithstanding the ligaturing of the carotid by one of our best surgeons, Dr. Roddick, the patient died.

Before passing altogether the anæsthetic properties of this fungus, I may mention that some years ago experiments were performed in Montreal, by Dr. Beers, by placing kittens in a chamber, to the outer surface of which there was a small iron box perforated beneath, and having a pipe opening into the box above. After freely distributing the fumes through the chamber, a kitten was put in. In six minutes it was insensible; remained thus for twenty minutes, having its ears clipped and otherwise treated without consciousness, and afterwards recovering and enjoying a good drink of milk.

If you inhale the lycoperdon through a hookah pipe, letting the fumes first pass through potash water, to clear them of carbonic acid, its effect is more lasting than chloroform. As a styptic in alveolar hæmorrhage, its effect is instantaneous. By removing anything in the way of thickened blood from the alveolar sockets, and opening the cellular tissue integument which invests them, bits of the fungus can be easily pressed in with the finger, and a piece of cork, spunk, lead or even cotton placed on top of it, and the jaws closed, and the patient kept quiet, cool and erect. It is wrong to let the patient lie down, or to give any alcoholic stimulant, as the object is to quiet the system.

I wish to draw attention to the fact that I have found the greatest virtue to lie in the genus lycoperdon bovista. The genus giganteum is the largest and easiest obtained; the bovista is small and scarce. The former is considered a distinct species, but the styptic properties of the bovista are much superior, containing a large proportion of phosphate of soda. It occurred to me to select the bovista, and medicate it with carbolic acid and camphor, by which means I have removed certain objections to it, and made it antiseptic as well as styptic.

I am indebted to Mr. Hoffman, of the Geological Survey of Canada, for assistance in obtaining the following analysis of the ash of bovista gigantæa. Mr. T. Nettleford, F. C. S., Englang, who made the analysis, speaking of the peculiar stalkless fungi growing close to the ground, infers that they collect the mineral matter from the soil:

| | Per cent. |
|---|---|
| Dry substance at 100 C . | 8 35 |
| Water . . . . . . | 91,65 |
| Ash . . . . . . | 0.571 |
| Ash on dry substance . . | 6.36 |

Analysis of the ash. Calculated on plans calculated on residue,

| | Per cent. | Per cent. |
|---|---|---|
| Resoluble residue in hydrochloric acid | 0.000 | |
| Alumina . . . . . . | 0.107 | 15.66 |
| Magnesia . . . . . . | 0.000 | 2.93 |
| Sulphuric acid (H. 2 So. 4) . . | 0.060 | 8.79 |
| Silica (Sig 2) . . . . . . | 0.003 | 0.44 |
| Lime (Cro.) mere traces . . . | | |
| Phosphate of soda . . . | 0.381 | 72.18 |

It is noticeable that phosphate of soda was once largely used to arrest hæmorrhage, and it appears that the styptic properties of the puff-ball is due to the excess of this substance.—*Dominion Dental Journal.*

